# Influenza virus infection increases ACE2 expression and shedding in human small airway epithelial cells

**DOI:** 10.1183/13993003.03988-2020

**Published:** 2021-07-01

**Authors:** Kelly S. Schweitzer, Taylor Crue, Jordan M. Nall, Daniel Foster, Satria Sajuthi, Kelly A. Correll, Mari Nakamura, Jamie L. Everman, Gregory P. Downey, Max A. Seibold, James P. Bridges, Karina A. Serban, Hong Wei Chu, Irina Petrache

**Affiliations:** 1 Dept of Medicine, National Jewish Health, Denver, CO, USA; 2 Division of Pulmonary Sciences and Critical Care Medicine, University of Colorado School of Medicine, Aurora, CO, USA; 3 Dept of Pediatrics, National Jewish Health, Denver, CO, USA; 4 Center for Genes, Environment and Health, National Jewish Health, Denver, CO, USA

## Abstract

**Background:**

Patients with coronavirus disease 2019 (COVID-19) caused by severe acute respiratory syndrome coronavirus 2 (SARS-CoV-2) demonstrate high rates of co-infection with respiratory viruses, including influenza A (IAV), suggesting pathogenic interactions.

**Methods:**

We investigated how IAV may increase the risk of COVID-19 lung disease, focusing on the receptor angiotensin-converting enzyme (ACE)2 and the protease TMPRSS2, which cooperate in the intracellular uptake of SARS-CoV-2.

**Results:**

We found, using single-cell RNA sequencing of distal human nondiseased lung homogenates, that at baseline, ACE2 is minimally expressed in basal, goblet, ciliated and secretory epithelial cells populating small airways. We focused on human small airway epithelial cells (SAECs), central to the pathogenesis of lung injury following viral infections. Primary SAECs from nondiseased donor lungs apically infected (at the air-liquid interface) with IAV (up to 3×10^5^ pfu; ~1 multiplicity of infection) markedly (eight-fold) boosted the expression of ACE2, paralleling that of STAT1, a transcription factor activated by viruses. IAV increased the apparent electrophoretic mobility of intracellular ACE2 and generated an ACE2 fragment (90 kDa) in apical secretions, suggesting cleavage of this receptor. In addition, IAV increased the expression of two proteases known to cleave ACE2, sheddase ADAM17 (TACE) and TMPRSS2 and increased the TMPRSS2 zymogen and its mature fragments, implicating proteolytic autoactivation.

**Conclusion:**

These results indicate that IAV amplifies the expression of molecules necessary for SARS-CoV-2 infection of the distal lung. Furthermore, post-translational changes in ACE2 by IAV may increase vulnerability to lung injury such as acute respiratory distress syndrome during viral co-infections. These findings support efforts in the prevention and treatment of influenza infections during the COVID-19 pandemic.

## Shareable abstract (@ERSpublications)

Influenza virus infection of cells lining the small airways increases the expression of molecules required for SARS-CoV-2 uptake in a manner that predicts increased severity of lung disease in those co-infected with influenza and coronaviruses https://bit.ly/3nu1WAo

## Introduction

Coronavirus disease 2019 (COVID-19), caused by the severe acute respiratory syndrome coronavirus 2 (SARS-CoV-2), is currently a major cause of death worldwide. Not all infected individuals develop symptoms, and most of those who do present with fever, cough and loss of smell indicative of respiratory tract infection involving mostly the upper and large/proximal airways. Patients in whom SARS-CoV-2 infects the lower small airways and lung parenchyma develop more serious disease such as pneumonia and acute respiratory distress syndrome (ARDS), which may culminate in respiratory failure and death [1, 2]. Notable from the beginning of the COVID-19 pandemic were the high rates of co-infection (20%) with other respiratory viruses [3–5], including influenza A virus (IAV) [6–10]. Accordingly, we set out to determine potential mechanisms by which IAV may predispose co-infection with SARS-CoV-2.

Angiotensin-converting enzyme 2 (ACE2) is the major receptor for SARS-CoV-2 entry into host cells. ACE2 is highly expressed on epithelial cells of the upper respiratory tract as well as gastrointestinal tract, explaining many of the early, less-severe manifestations of COVID-19. In turn, more severe manifestations of COVID-19 develop when SARS-CoV-2 infects epithelial cells in the distal lung including the small airways and alveoli [11] lined by small airway epithelial cells (SAECs) and alveolar type II (ATII) and type I (ATI) cells [12]. SAECs play an essential role in the structural integrity of the lung parenchyma and form a last line of defence of the alveoli against environmental insults. The expression and regulation of ACE2 in SAEC is not known, although recent publications suggest low levels of expression of ACE2 in the distal lung. However, IAV infection of primary human ATII cells increased *ACE2* mRNA expression [13], and the *ACE2* gene expression is regulated by interferons (IFNs) [14], a family of cytokines activated by multiple respiratory viruses. These findings suggest that IAV may increase the abundance of the receptor for SARS-CoV-2 in human SAECs.

To facilitate SARS-CoV-2 uptake *via* the ACE2 receptor, the activation of the serine protease TMPRSS2 is essential, through a process that involves ACE2 cleavage. Whereas there is abundant evidence that TMPRSS2 is also essential for IAV virulence by cleaving haemagglutinin, the impact of IAV on TMPRSS2 is less clear.

While ACE2 cleavage by TMPRSS2 aids in virus uptake, the loss of ACE2 from plasma membranes severely affects the homeostatic enzymatic function of ACE2, specifically the conversion of angiotensin II to angiotensin. It is well established that loss of ACE2 from the cell surface increases the risk of severe acute lung injury [15] in models of IAV and SARS-CoV-1 [16, 17]. A well-established mechanism of ACE2 inactivation is *via* proteolytic cleavage by the tumour necrosis factor-α converting enzyme (TACE), known as a disintegrin and metalloproteinase domain-containing protein (ADAM)17.

Using primary human SAECs grown at the air-liquid interface, we demonstrate that IAV has a major impact on both ACE2 and TMPRSS2 expression and post-translational modifications, in a fashion that indicates that co-infection with IAV may increase the risk of developing severe lung disease from SARS-CoV-2.

## Materials and methods

### Reagents

All reagents were purchased from Sigma-Aldrich (St Louis, MO, USA) unless otherwise stated.

### Human subjects

Lungs from de-identified organ donors that were not used for transplantation were donated for medical research through the National Disease Research Interchange (Philadelphia, PA, USA), the International Institute for the Advancement of Medicine (Edison, NJ, USA) or the Donor Alliance (Denver, CO, USA). The committee for the protection of human subjects at National Jewish Health (Denver, CO) deemed this research as human research-exempt. We used lungs from donors with a diagnosis of brain death and without a history of lung disease, who were lifelong nonsmokers. At the time of lung harvest for donation, the donor had to have absence of lung injury as indicated by an arterial oxygen tension/inspiratory oxygen fraction ratio >300, a chest radiograph without changes to indicate an acute process and mechanical ventilation of <5 days. The sex, age and race were variable and were not selection criteria; basic demographic information is detailed in table 1.

### Single-cell RNA sequencing of human lungs

Cells were isolated from three human lungs from de-identified organ donors whose lungs were not suitable for transplantation. The Human Research Protection Program at National Jewish Health deemed this research as nonhuman subject research. The lungs were perfused, lavaged and digested with elastase, as described previously [18]. Lungs were minced and cells of haematopoietic origin were depleted using anti-CD45-coated magnetic beads (Miltenyi Biotech, Bergisch Gladbach, Germany) and magnetic-activated cell sorting [19]. The cell pellet obtained by centrifugation was resuspended in 1 mL cold PBS+dithiothreitol, centrifuged (225×*g*; 4°C; 5 min) and washed twice with cold PBS. The final cell pellet was resuspended in PBS with 0.04% bovine serum albumin for single-cell gene expression profiling with the 10X Genomics system. Sample capture, cDNA synthesis and library preparation were performed using protocols and reagents for 10X Genomics Chromium Single Cell 3′ v3 kit [20]. Single-cell libraries were pooled for sequencing on an Illumina NovaSeq 6000 Reads were aligned to the hg19 genome assembly and processed according to the Drop-Seq Computational Protocol v2.0 (www.github.com/broadinstitute/Drop-seq). Initial processing of 10X scRNA-seq data, including cell demultiplexing, alignment to the human genome GRCh38 and unique molecular identifier (UMI)-based quantification was performed with Cell Ranger (version 3.0). To ensure that high-quality cells were used for downstream analysis, we removed cells with <100 genes detected and cells with >25% mitochondrial reads. Additionally, to remove possible doublets, we removed cells with >100 000 UMIs. For gene filtering, we removed lowly expressed genes (detected in fewer than four cells). Using the filtering described, we obtained a dataset consisting of 17 581 cells and 23 198 genes. Prior to clustering, we performed normalisation using SCTransform and integration of datasets from four whole lung samples using a mutual nearest neighbour based approach. Clustering analysis was performed on the top 30 principal components using the Louvain algorithm. Visualisation of single-cell expression profiles into a two-dimensional map was computed using the uniform manifold approximation and projection technique. Differential expression analysis between cell clusters was conducted on the count per million normalised count matrix using the FindMarkers function with default options. All the analyses were carried out with R Seurat package version 3.0.3.

### SAEC isolation and culture

Small airway (<2 mm diameter) epithelial cells were collected from the distal lung using a 2 mm bronchoscopy brush (Conmed, Greenwood Village, CO, USA) and placed in sterile PBS. Cells were isolated by centrifugation, resuspended in PBS, counted and plated onto an irradiated NIH 3T3 fibroblast feeder layer in F-media. Once visible colonies had formed (7–10 days), they were removed with 0.25% trypsin (Corning, Corning, NY, USA; cat # 25-053-CI) and plated on double collagen-coated 12-well transwell tissue culture inserts (Advanced BioMatrix, Carlsbad, CA, USA; cat # 5005 and Corning; cat # 3460). Cells were cultured in Pneumacult ALI media (StemCell Technologies, Vancouver, Canada), as reported previously [21] and maintained at 5% carbon dioxide at 37°C.

### IAV exposure of SAECs cultured at the air—liquid interface

After 14–21 days of differentiation at the air—liquid interface, SAECs were infected at the apical surface with IAV (A/California/07/2009 H1N1, pdm09) using a low-dose 3×10^2^ pfu·transwell^−1^ (estimated 0.001 multiplicity of infection (MOI)), a high-dose 3×10^5^ pfu·transwell^−1^ (~1 MOI) or left uninfected (control), in 250 μL culture medium. Following 2 h incubation, the virus-containing media was removed, cells were washed, and incubated with 250 μL of cell culture medium. Supernatant and cells were collected at 48 or 72 h post- IAV infection. Cell lysates from IAV-infected or uninfected SAECs were loaded onto gels for Western blotting along with similarly infected cells that were pre-exposed to e-cigarette vapour, which, at the concentrations used, had no effect on ACE2 or TMPRSS2 protein.

### E-cigarette vapour exposure of SAECs at the air—liquid interface

SAECs were exposed at the air—liquid interface to e-cigarette vapour (Juul, San Fransisco, CA, USA) using a Vitrocell VC1 smoke delivery system using the Coresta Recommended Method (CRM) #81 smoke profile (CRM81). SAECs were exposed twice, 2 h apart, apically to Juul using puff volume of 55 mL, puff duration of 3 s, exhaust time of 8 s, puff frequency of 30 s for a total of 10 puffs. Cells were then incubated at 37°C for 24 h, followed by infection with IAV as described earlier.

### IAV exposure of SAECs submerged in culture media

For select experiments, primary human SAECs (cultured to confluence up to passage 4) were exposed to IAV 0.5×10^5^ pfu·transwell^−1^ (estimated ~0.5 MOI, low dose) or 1×10^5^ pfu·transwell^−1^ (~1 MOI, high dose), for 2 h, while still submerged in B-ALI media (Lifeline Cell Technologies, Frederick, MD, USA). Following four washes with PBS, cells were incubated with regular culture media without virus and supernatants were collected 48 h later.

### Quantitative reverse transcription-PCR

Total mRNA was isolated using the RNAeasy Micro Kit (Qiagen, Germantown, MD, USA) or the GenCatch Total RNA Miniprep Kit following manufacturer’s instructions. cDNA was transcribed (High-Capacity cDNA Reverse Transcription Kit; ThermoFisher, Waltham, MA, USA) and quantitative (q) PCR was then performed on the cDNA (StepOnePlus System; ThermoFisher) using Taqman Universal PCR Master Mix (ThermoFisher) and probes specific for human *ACE2* (ThermoFisher; Hs01085333_m1, lot # 1864532), human *TMPRSS2* (ThermoFisher; Hs00237175_m1), human ADAM17 (ThermoFisher; Hs01041915_m1), and human *STAT1* (ThermoFisher; Hs01013996_m1). Relative fold expression was calculated using the double delta Ct method with endogenous control eukaryotic *18S* rRNA as reference gene (ThermoFisher; Hs99999901_s1, lot # 1505039).

### Concentration of cell supernatants

Cell supernatants were collected form the apical surface of SAECs grown at the air—liquid interface that were incubated with apically added virus-containing media for the indicated time. These supernatants (150 μL per well) were concentrated using Amicon Ultra Centrifugal Filters 3K (EMD Millipore; cat # UFC500396, lot # R1MA18999) or Pierce Protein Concentrators PES 50K (ThermoScientific; cat # 88504, lot # VH310324), following the respective manufacturer’s specifications. Concentrated supernatants were mixed with Laemmli buffer and were loaded onto gels for Western blotting.

### Immunoblotting by Western blotting

Cell pellets were resuspended in standard RIPA buffer (cat # R0278, lot # SLBL7395V) on ice for 30 min, collected by centrifugation, and quantified using standard BCA assay. Proteins were resolved by 4–20% gradient PAGE and transferred using semi-dry transfer apparatus (Bio-Rad, Hercules, CA) onto Immobilon-P PVDF membranes (cat # IPVH00010). Membranes were blocked in Pierce Protein-Free T20 (TBS) Blocking Buffer (Thermo Fisher, cat # 37571) and washed using TBS with 0.1% Tween-20. We probed membranes with either polyclonal # AF933 (lot # HOK 0320032; 1:200; R&D Systems, Minneapolis, MN) or polyclonal # 21115-1-ap (1:2000; ProteinTech) anti-ACE2 antibodies. Other antibodies used were anti-TMPRSS2 # 14437-1-AP (1:500; ProteinTech), anti-vinculin # CP74, or anti-β-actin # A5441. Secondary antibodies were HRP-conjugated anti-goat # 305-035-003 (lot # 145804; Jackson ImmunoResearch, West Grove, PA); anti-mouse # NA931V (lot # 9715064, GE Healthcare, Chicago, IL); and anti-rabbit # NA9340V (lot # 10997954, GE Healthcare).

### Validation of ACE2 antibodies

We used recombinant human ACE2 (R&D Systems, cat # 933-ZN, lot # FIU0620041) in buffer (25 mM Tris (pH 10.0), 2.5 μM ZnCl_2_, 0.005% Brij-35) and human embryonic kidney (HEK)293T cells overexpressing human (h)ACE2. The latter was obtained as follows: hACE2 cDNA was PCR-amplified from plasmid # 1786 (Addgene) (a gift from Hyeryun Choe, The Scripps Research Institute, Jupiter, FL, USA) [22] with a flanking Kpn1 and Kozak site in the sense primer and a Xba1 site in the anti-sense primer (sense: GGTACCGCCACCATGTCAAGCTCTTCCTGGCTCC; anti-sense: TCTAGACTAAAAGGAGGT CTGAACATCATCAGTG). The amplified insert was cloned in a vector containing a pCASI promoter (pCASI-MCS-WPRE) and sequenced. Following sequence confirmation, the pACASI-hACE2-WPRE vector was transiently transfected into HEK293T cells (24 h), then cells were lysed in standard radioimmunoprecipitation assay (RIPA) buffer containing protease inhibitors, sonicated and stored at −20°C.

### Statistical analyses

Statistical testing was performed using Prism v.6 (GraphPad Software, San Diego, CA, USA). One-way ANOVA with Sidak’s multiple comparisons test was used to compare differences in means between groups when more than two groups are present. A t-test (two-tailed Mann—Whitney test) was applied to log_2_--transformed data comparisons of two groups. A p-value <0.05 was considered significant.

## Results

### ACE2 and TMPRSS2 expression in human SAECs in situ in distal lung

**FIGURE 1:**
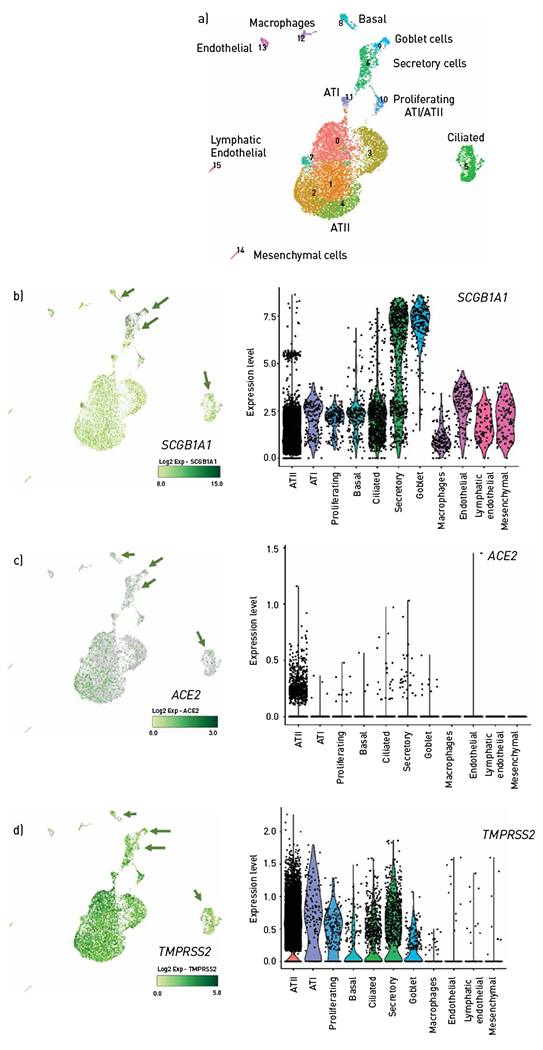
*ACE2* and *TMPRSS2* mRNA expression in human lungs. a) Uniform manifold approximation and projection projections (UMAP) of the single-cell RNA data obtained from human donor lungs from individuals without lung disease (n=3). Note colour-coded populations of specific cell types, as noted, including airway epithelial cell types identified through unsupervised clustering. b–d) UMAP and violin plots of normalised expression of *SCGB1A1*, a marker of b) airway epithelial cells, c) *ACE2* and d) *TMPRSS2* in the identified cell type; colocalisation with *SCB1A1* is indicated by arrows. ATI: alveolar type I cells; ATII: alveolar type II cells.

Using single-cell RNA-seq assessment of whole-lung homogenates from four donor lungs sorted for CD45^−^ cell population, we determined the expression of *ACE2* and *TMPRSS2* in SAECs. Utilising a shared nearest neighbour-based clustering approach on the expression profiles of >10000 cells, we identified ATII cells; ATI cells; proliferating cells; ciliated, early ciliating, basal, goblet secretory airway epithelial cells; macrophages; and mesenchymal, endothelial and lymphatic endothelial cells (figure 1a, supplementary material). SAECs (<2 mm) express *SCGB1A1* and include basal cells, goblet, secretory and early ciliating cells (figure 1b). Consistent with previous reports, cell types comprising small epithelial cells in the distal human lung express both *ACE2* (at low levels in healthy lungs) and *TMPRSS2* mRNA (figure 1c,d).

### Effect of IAV on ACE2 mRNA levels in human SAECs

**FIGURE 2:**
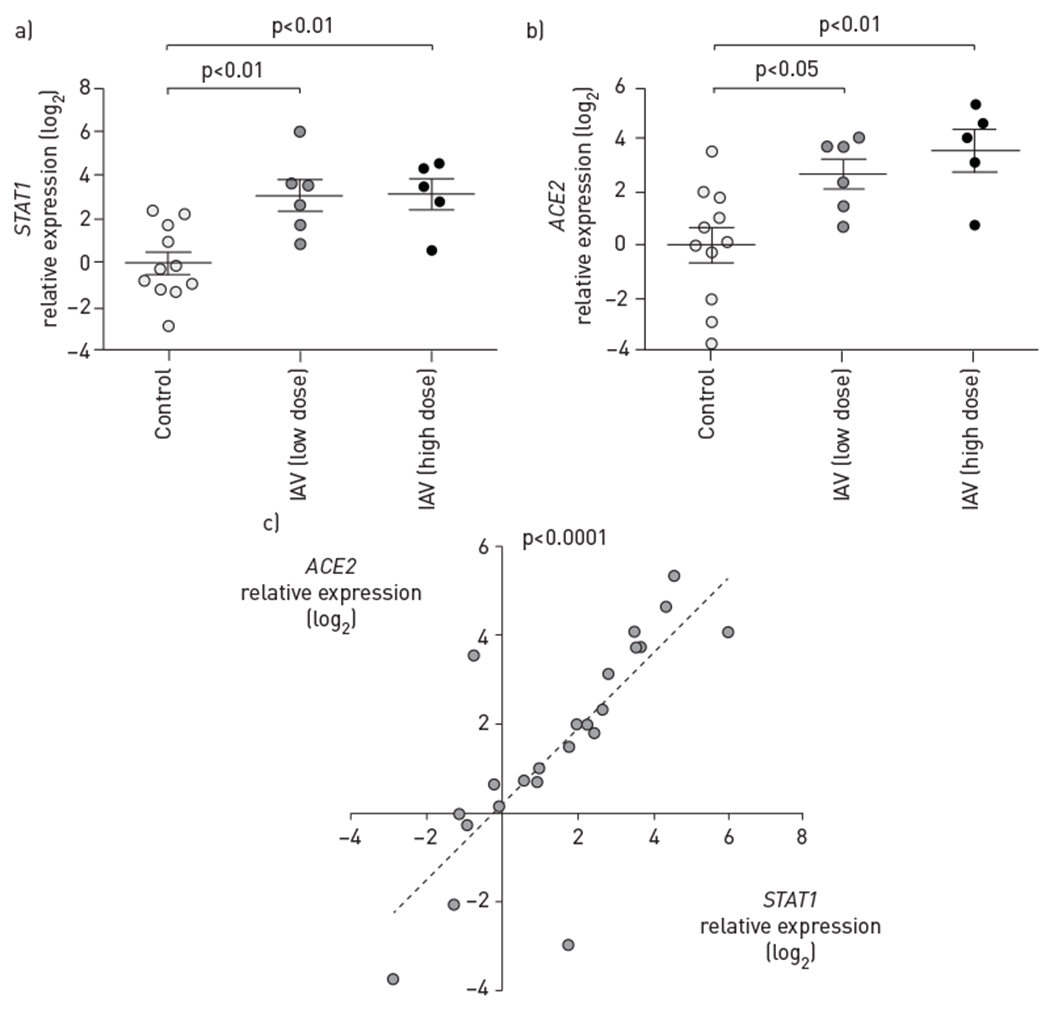
Effect of influenza A virus (IAV) infection on *ACE2* transcription in small airway epithelial cells (SAECs). Relative levels of a) *STAT1* and b) *ACE2* mRNA measured using reverse transcription quantitative PCR using 18S rRNA as control, expressed as log_2_ of 2^−ΔΔCT^, following infection of SAECs at the air—liquid interface with IAV low dose (H1N1 pdm09 virus, 3×10^2^ pfu·transwell^−1^, 72 h) or high dose (H1N1 pdm09 virus, 3×10^5^ pfu·transwell^−1^, 48 h). Each data point represents an independent experiment from n=6 and n=4 (low and high dose, respectively). Data are presented as mean±SEM; one-way ANOVA—Sidak multiple comparison test. c) Correlation by linear regression between *STAT1* and *ACE2* expression levels in SAECs; each data point represents an independent IAV-infected or -uninfected experimental condition. Pearson correlation coefficient r=0.78; R^2^=0.61, p<0.0001.

We next used a well-established infection model [23–26], in which IAV is delivered apically to primary human SAECs cultured at the air—liquid interface. As expected, low (H1N1 pdm09 virus, 3×10^2^ pfu·transwell^−1^) or high dose (H1N1 pdm09 virus, 3×10^5^ pfu·transwell^−1^) of IAV increased *STAT1* mRNA levels, as measured by reverse transcription (RT)-qPCR (figure 2a). In addition, both doses of IAV markedly and significantly increased *ACE2* expression in SAECs when compared to noninfected cells, by 8.7-fold and 13.9-fold, respectively (figure 2b). Consistent with virus-induced activation of *ACE2* transcription, there was a significant correlation between *STAT1* and *ACE2* expression levels in SAECs (figure 2c).

### Effect of IAV on ACE2 protein in human SAECs

**FIGURE 3:**
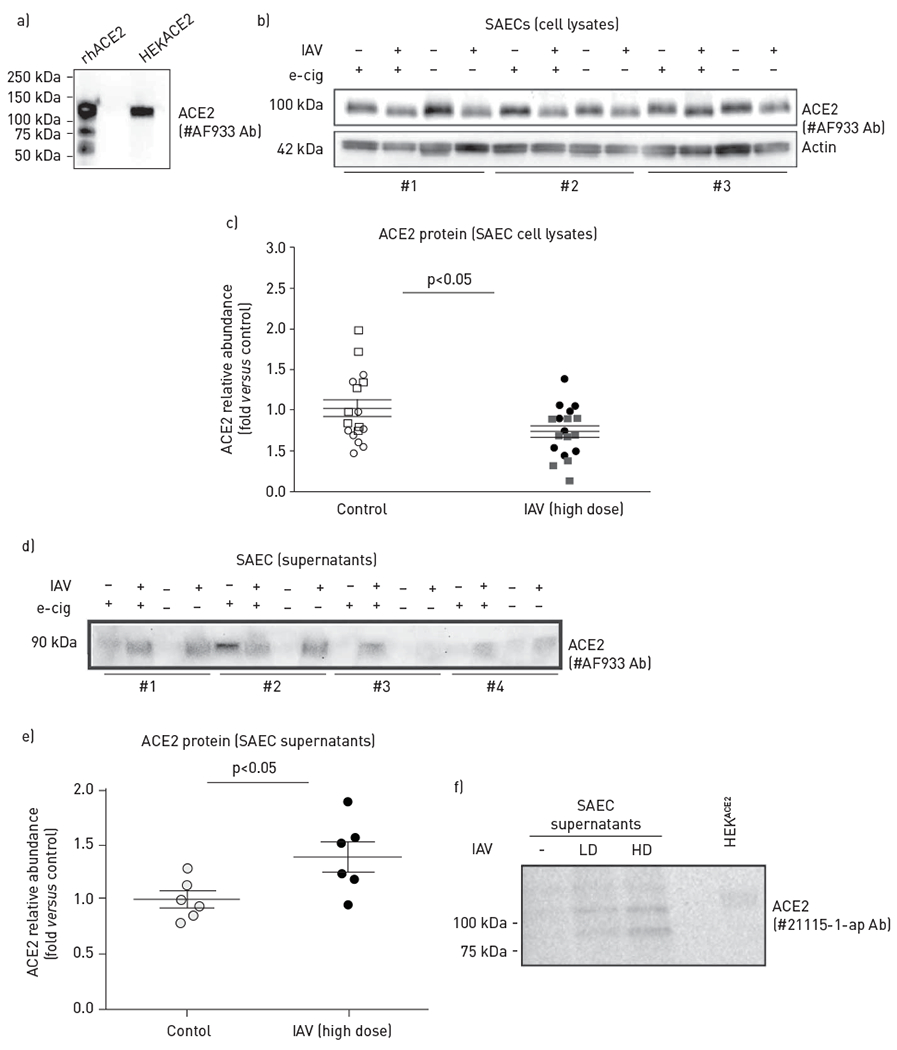
Effect of influenza A virus (IAV) on angiotensin-converting enzyme (ACE)2 protein levels and shedding in small airway epithelial cells (SAECs). a) Validation of ACE2 antibody (#AF933) using immunoblotting of recombinant human (rh)ACE2 protein and whole-cell lysate (1.6 μg) of human embryonic kidney (HEK)293T cells induced to overexpress human ACE2 (HEK^ACE2^). b,c) Intracellular ACE2 protein in SAECs infected at the air—liquid interface with IAV (+) (H1N1 pdm09 virus, 3×10^5^ pfu·transwell^−1^, 48 h) compared to uninfected cells (−), with indicated pre-exposure (+) to e-cigarette vapour (e-cig); b) ACE2 was detected by immunoblotting with the polyclonal antibody #AF933 and c) quantified by densitometry after normalisation to actin levels used as loading control. Cell lysates obtained from distinct donors (#1–3) are noted. d,e) Released ACE2 protein in apical supernatants (normalised by volume) of SAECs infected with IAV (+) at the air—liquid interface, with the indicated pre-exposure (+) to e-cig; ACE2 d) detected by immunoblotting with #AF933 antibody and e) quantified by densitometry. Supernatants obtained from cells from distinct donors (#1–4) are noted. f) Released ACE2 protein in apical supernatants from SAECs infected while submerged in culture media with lower dose (LD; 0.5×10^5^ pfu·transwell^−1^) or higher dose (HD; 1.0×10^5^ pfu·transwell^−1^) IAV for 48 h; ACE2 was detected by immunoblotting (with antibody # 21115-1-ap). Cell lysate (1.6 μg) from HEK^ACE2^ was used as control. Graphs show individual data points from independent experiments, mean±SEM; t-test.

We evaluated ACE2 protein levels in cells and in the apical media of SAECs infected with IAV (3×10^2^ or 3×10^5^ pfu·transwell^−1^) using Western blotting. We first used the polyclonal antibody #AF933, which we validated using recombinant human ACE2 protein and cell lysates from HEK cells overexpressing human ACE2 (figure 3a). Compared to uninfected cells, where ACE2 exhibited an expected electrophoretic mobility of ~110 kDa, cell lysates of IAV-infected SAECs showed a minor but consistent shift (increase) in ACE2 electrophoretic mobility (figure 3b), together with an overall modest but significant decrease in ACE2 levels, measured by densitometry (figure 3c). This suggested post-translational changes in ACE2 that could include cleavage with intracellular loss of the protein. We then evaluated if there was any secretion of ACE2 at the apical surface of SAECs. Supernatants were obtained by collecting equal volumes of the media applied at the apical side of SAECs at the air—liquid interface at 48 h following IAV exposure. In the apical media from IAV-infected cells we noted a consistent presence of an ACE2 peptide with an electrophoretic mobility of ~90 kDa (figure 3d), suggesting the release of an ACE2 fragment from cells. Quantification by densitometry showed a significant increase in ACE2 in the apical supernatants of SAECs infected with IAV (figure 3e). In this experiment, we also investigated the effect of e-cigarette vape exposure, which may modify SAEC secretory function, and the antiviral responses of nasal epithelial cells [21, 27]. We did not detect a consistent effect of e-cigarettes on ACE2 on either IAV infected or noninfected cells, although in some e-cigarette-exposed cells we noted the release of ACE2 fragment in the apical media (figure 3b,d). As a complementary approach, we next tested a distinct polyclonal anti-ACE2 antibody and ACE2-overexpressig HEK cells as positive control and probed supernatants of SAECs submerged in media. We confirmed a dose effect of IAV on ACE2 protein fragments release by SAECs, even in culture conditions that precede full differentiation achieved at the air—liquid interface (figure 3f). The lower molecular weight of the ACE2 peptide found in supernatants compared to that in cell lysates indicate that ACE2 may undergo proteolytic cleavage and release from the plasma membrane in response to IAV infection of SAECs.

### Effect of IAV on proteases capable of cleaving ACE2 in human SAECs

**FIGURE 4:**
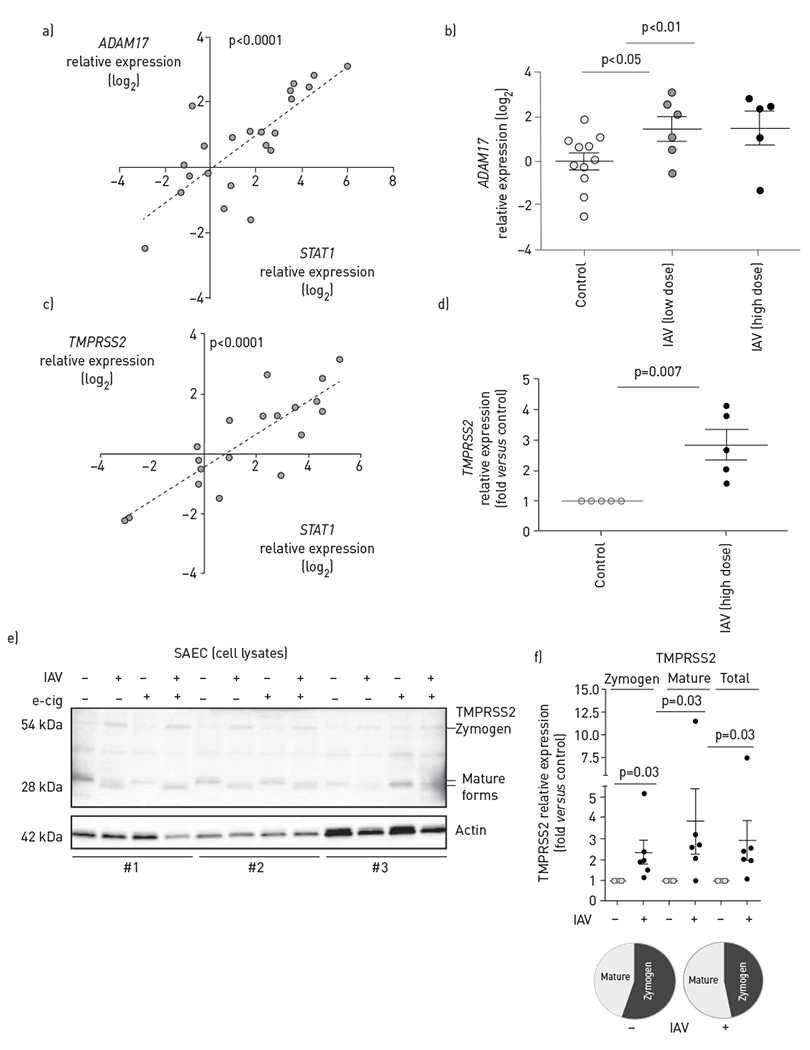
Effect of influenza A virus (IAV) on *ADAM17* and *TMPRSS2* levels in small airway epithelial cells (SAECs). a–d) Expression levels of indicated proteases measured by reverse transcription (RT) quantitative PCR in SAECs uninfected or infected at the air—liquid interface with IAV at low dose (H1N1 pdm09 virus, 3×10^2^ pfu·transwell^−1^, 72 h) or high dose (H1N1 pdm09, 3×10^5^ pfu·transwell^−1^, 48 h); each data point represents an independent experimental condition. a) Correlation between *STAT1* and *ADAM17* expression levels in SAECs by linear regression; Pearson correlation coefficient R^2^=0.59, p<0.0001; b) *ADAM17* mRNA levels; c) correlation between *STAT1* and *TMPRSS2* expression levels in SAECs by linear regression; Pearson correlation coefficient R^2^=0.70, p<0.0001; d) *TMPRSS2* mRNA levels. e) TMPRSS2 protein (zymogen and mature forms) in SAECs infected with IAV (+) compared to uninfected cells (−), with indicated pre-exposure (+) to e-cigarette vapour (e-cig) detected by Western blotting; cell lysates obtained from distinct donors (#1–3) are noted; f) relative changes in zymogen, mature and total TMPRSS2 levels induced by IAV were quantified by densitometry relative to uninfected controls, using actin loading control for normalisation. Individual data points indicate independent experiments; mean±SEM, Wilcoxon signed rank test. Pie charts indicating relative levels of mature forms of total TMPRSS2 protein.

We next investigated the effect of IAV on two of the proteases known to cleave ACE2, the sheddase ADAM17 (TACE) and the serine protease TMPRSS2. Both *ADAM17* and *TMPRSS2* contain binding sites for the transcription factor STAT1 [28, 29]. Indeed, we found a strong correlation between *STAT1* and *ADAM17* expression levels in SAECs (figure 4a). Compared to uninfected cells, IAV infection upregulated *ADAM17* in SAECs, as measured by RT-qPCR (figure 4b). *TMPRSS2* was similarly associated with *STAT1* in SAECs and was also significantly increased by IAV infection (3×10^5^ pfu·transwell^−1^) (figure 4c, d). Next, we investigated the effect of IAV infection on TMPRSS2 protein, which is synthesised as a zymogen that undergoes proteolytic autoactivation necessary for its serine protease function. IAV increased the abundance of both TMPRSS2 zymogen (54 kDa) and especially that of the lower molecular weight mature TMPRSS2 fragments of ~35 kDa and especially that of ~28 kDa (figure 4e,f).

## Discussion

Our study indicates that infection of epithelial cells lining the human distal airways with IAV markedly increases the transcription of molecules necessary for SARS-CoV-2 uptake. Furthermore, IAV infection may cause post-transcriptional changes of ACE2 that may decrease its convertase activity, which in turn has been associated with increased severity of acute lung injury from respiratory viral infections, including influenza and SARS-coronaviruses.

To our knowledge, this is the first report of ACE2 and TMPRSS2 regulation by IAV in human SAECs in a model that mimics lung exposures to respiratory viruses. The cell type at the core of our study is very important in this context, since these cells express little ACE2 and TMPRSS2 at baseline and may be shielded from distal lung (severe) infection with SARS-CoV-2 in the absence of IAV infection. Moreover, we believe that is the first report, in any cell type, demonstrating that IAV modifies both *ACE2* and *TMPRSS2* mRNA expression and post-transcriptional regulation in a manner consistent with priming for more severe COVID-19 manifestations.

The ACE2 receptor binds the spike protein (S-protein) on the surface of SARS-CoV-2, facilitating the virus attachment and entry into the host cell; conversely, administration of full-length ACE2 decorates SARS-CoV-2, reducing cell uptake and infection [30]. *ACE2* is expressed in multiple lung cell types, including ATII cells, airway epithelial cells, macrophages, endothelial cells and fibroblasts [11, 31], but the baseline expression levels in these cells varies. In COVID-19, SARS-CoV-2 first infects upper and large airway epithelial cells known to have higher expression of ACE2 [32, 33]. In the vast majority of exposed individuals, the infection remains limited to the upper airways, causing asymptomatic or mild respiratory disease. When SARS-CoV-2 infects cells in the distal airways and airspaces such as ATII cells, which have a low baseline ACE2 expression [32], the disease has more severe manifestations, due to inflammation and injury of gas exchange areas of the lung. Therefore, infection with SARS-CoV-2 appears to parallel the levels of ACE2 expression [34], although the expression of TMPRSS2 and other receptors are also likely to contribute to SARS-CoV-2 infectivity [33].

We performed our own evaluation of single-cell RNAseq data in human nondiseased lungs focusing on the distal lungs, which distinguished our study from prior work. Although the donors were young males, our data corroborated published results that used a broader demographic. Additionally, the pattern of expression of ACE2 and TMPRSS noted in whole-lung homogenates was recapitulated in SAECs *in situ*, showing that the study of these primary human cells in cultures is relevant. Moreover, the ACE2 expression in SAECs isolated from lungs of both male and female donors with a broad age range was consistent with that found in single-cell experiments. Prior to this report, airway epithelial cells isolated from small airways have been infrequently used to study the pathogenicity of respiratory viruses, most studies having relied on human bronchial epithelial cells from larger airways either differentiated at the air—liquid interface [23] or guided into forming organoids [35]. The fact that IAV increased *ACE2* expression is not surprising, since viral infections can increase *ACE2* mRNA transcription *via* transcription factors such as STAT1 as part of the IFN signalling response [14, 36]. The significant correlation we measured between *STAT1* and *ACE2* mRNA levels suggests that a similar IFN-induced response is likely to account for the increase in *ACE2* in response to IAV infection of SAECs. The boosting effect of IAV infection on *ACE2* expression in SAECs coupled with its potential to increases *ACE2* in ATII [13] suggests that the influenza infection may be a risk factor for distal lung co-infection with SARS-CoV-2. The clinical implication of such events is that infection with SARS-CoV-2 of small-airway and alveolar epithelium causes respiratory compromise such as pneumonia and ARDS. Furthermore, insufficient or maladaptive repair of the viral-injured distal lung epithelium may contribute to the development of chronic diseases such as interstitial pulmonary fibrosis or emphysema, feared chronic sequelae of COVID-19 pneumonia.

Following the binding of SARS-CoV-2 virus to ACE2, proteases such as TMPRSS2, facilitate viral entry into host cells [37–39]. TMPRSS2 is an androgen-regulated serine protease with transmembrane, receptor class A, scavenger receptor and protease domains. Expressed at the surface of epithelial cells, including those in the lung [40], TMPRSS2 has been known to increase the virulence of IAV infection by cleaving viral haemagglutinin [41, 42]. However, little is known about the regulation of TMPRSS2 by IAV itself. To our knowledge, we found for the first time that IAV infection increases both the transcription and protein levels of TMPRSS2. We noted the increase of multiple TMPRSS2 forms, corresponding to the N-glycosylated zymogen (~60 kDa), and the two mature forms (~32 kDa and ~28 kDa), typically generated from auto-activation of the protease [43–45]. Although we did not measure TMPRSS2 activity, these post-translational changes caused by IAV, with increases in mature peptides, may reflect proteolytic autoactivation of the TMPRSS2 zymogen. Alternatively, oxidative stress, which can accompany IAV infections, impacts TMPRSS2 localisation at the plasma membrane and may also cause post-translational modifications of disulfide bonds [46]. Given the uncontested role of TMPRSS2 in the pathogenicity of both IAV and SARS-CoV-2, our data implicates TMPRSS2 in the severity of lung co-infections with IAV and SARS-CoV-2.

SARS-CoV-2 infection may not only increase *ACE2* transcription (*via* IFN response), but may concomitantly reduce the availability of catalytically active ACE2 at plasma membranes by inducing cleavage with shedding extracellular or by enhancing the intracellular internalisation of ACE2 [16, 47]. The shedding of ACE2 is mediated by the protease ADAM17 (TACE) [48, 49]; although in Vero E6 cells this enhanced SARS-CoV uptake, and the role of ADAM17 in this function remains controversial [39, 45, 48, 50]. In turn, there is robust evidence that ACE2 cleavage from cell surface, by reducing its convertase activity, increases angiotensin II with subsequent increased vascular permeability and lung oedema [16], and reconstitution of ACE2 by administration of full-length peptide reduces lung injury [16, 17, 30, 51]. ADAM17 is activated by inflammatory mediators at the surface of multiple cell types, including lung epithelial cells [52]. We found that concomitantly with upregulation of *ADAM17* expression, IAV infection of SAECs decreases intracellular levels of ACE2, changes the electrophoretic mobility of intracellular ACE2 (suggesting that the remaining intracellular ACE2 is of a lower molecular weight) and induces the appearance of an ACE2 fragment in media applied apically to cells. A similar change in intracellular ACE2 was reported in a study following IAV infection of human nasopharyngeal carcinoma cell line CNE-2Z and embryonic kidney cell line 293T. However, the shift was attributed to ACE2 cleavage by IAV proteases and proteasomal degradation, rather than shedding, as ACE2 was not detected in supernatants [53]. The marked increases in shedding of ACE2 from SAECs infected with IAV (at concentrations similar to those used by Liu *et al*. [53]) suggests that IAV effects may be cell type-specific, although we did not exclude the possibility that IAV proteases are also implicated in the ACE2 post-translational changes in our study. Pre-exposure of cells to e-cigarette did not alter (augment) their response to IAV, although it tended to increase ACE2 fragment secretion into apical supernatant, suggesting either a maximal effect of IAV, or that longer exposure to e-cigarette vaping than utilised in this study may be needed induce ACE2.

By decreasing catalytically active ACE2 in human SAECs, IAV may worsen the acute lung injury induced by SARS-CoV-2. Several clinical and pre-clinical investigations support this concept: IAV infection is a recognised risk factors for “typical” ARDS [54]; plasma of patients with severe influenza have increased circulating ACE2 levels [55]; and mouse models of influenza-induced lung injury are worsened in Ace2-knockout animals [55] and alleviated by administration of recombinant ACE2 [17].

**FIGURE 5:**
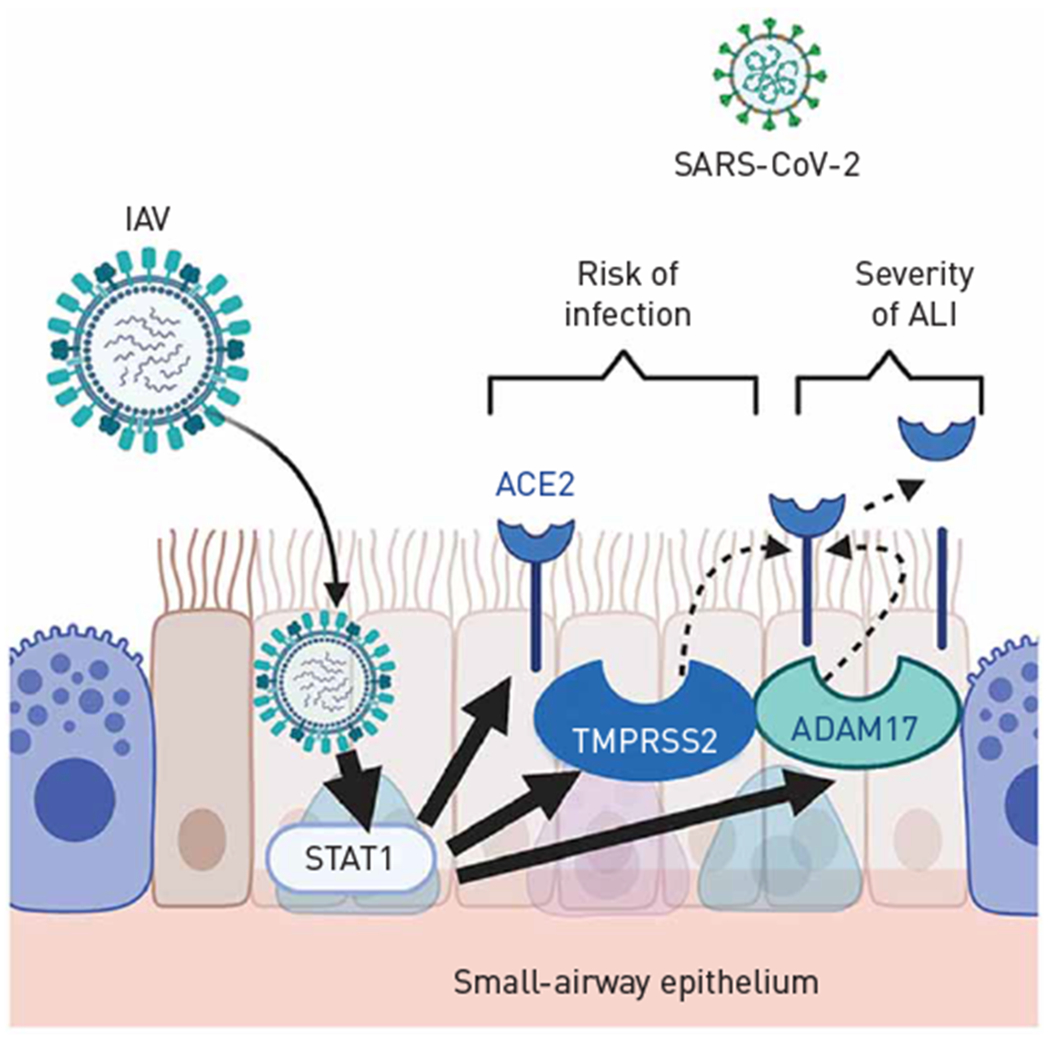
Schematic showing the interpretation of results in the context of putative severe acute respiratory syndrome coronavirus 2 (SARS-CoV-2) co-infection and resultant lung injury. IAV: influenza A virus; ALI: acute lung injury; ACE2: angiotensin-converting enzyme 2.

Although a limitation of our study is the lack of directly testing of the effect of IAV on SARS-CoV-2 infection, our findings provide significant evidence of IAV-mediated regulation of proteases and receptors in a pattern that predicts increased SARS-CoV-2 co-infectivity and worse lung injury following viral infection. Given the predicted seasonality of respiratory viruses, there is concern about coincidental waves of SARS-CoV-2 infection with flu seasons [56]. Our study provides evidence that IAV infection may be a risk factor for enhanced infectivity of the human lower airways and for increased severity of lung disease induced by SARS-CoV-2 (schematic in figure 5). These findings may contribute to increased impetus to encourage influenza virus vaccination of those at high risk of COVID-19.

## Supplementary Material

Suppl data in Excel

## Figures and Tables

**TABLE 1.** Demographic information related to donor lungs used for small airway epithelial cell studies

Subjects	Age years	Sex
1	76	Female

2	59	Female

3	40	Male

4^#^	18	Male

5	19	Female

6	44	Male

7	57	Male

All subjects were nonsmokers.

# : this subject had a history of vaping.
